# Modular Coils with Low Hydrogen Content Especially for MRI of Dry Solids

**DOI:** 10.1371/journal.pone.0139763

**Published:** 2015-10-23

**Authors:** Timon Eichhorn, Ute Ludwig, Elmar Fischer, Jens Gröbner, Michael Göpper, Anne-Katrin Eisenbeiss, Tabea Flügge, Jürgen Hennig, Dominik von Elverfeldt, Jan-Bernd Hövener

**Affiliations:** 1 Medical Physics, Department of Radiology, University Medical Center Freiburg, Freiburg, Germany; 2 Biological Anthropology, University Medical Center Freiburg, Freiburg, Germany; 3 Department of Craniomaxillofacial Surgery, University Medical Center Freiburg, Freiburg, Germany; University of California San Francisco, UNITED STATES

## Abstract

**Introduction:**

Recent advances have enabled fast magnetic resonance imaging (MRI) of solid materials. This development has opened up new applications for MRI, but, at the same time, uncovered new challenges. Previously, MRI-invisible materials like the housing of MRI detection coils are now readily depicted and either cause artifacts or lead to a decreased image resolution. In this contribution, we present versatile, multi-nuclear single and dual-tune MRI coils that stand out by (1) a low hydrogen content for high-resolution MRI of dry solids without artifacts; (2) a modular approach with exchangeable inductors of variable volumes to optimally enclose the given object; (3) low cost and low manufacturing effort that is associated with the modular approach; (4) accurate sample placement in the coil outside of the bore, and (5) a wide, single- or dual-tune frequency range that covers several nuclei and enables multinuclear MRI without moving the sample.

**Materials and Methods:**

The inductors of the coils were constructed from self-supporting copper sheets to avoid all plastic materials within or around the resonator. The components that were mounted at a distance from the inductor, including the circuit board, coaxial cable and holder were manufactured from polytetrafluoroethylene.

**Results and Conclusion:**

Residual hydrogen signal was sufficiently well suppressed to allow ^1^H-MRI of dry solids with a minimum field of view that was smaller than the sensitive volume of the coil. The SNR was found to be comparable but somewhat lower with respect to commercial, proton-rich quadrature coils, and higher with respect to a linearly-polarized commercial coil. The potential of the setup presented was exemplified by ^1^H / ^23^Na high-resolution zero echo time (ZTE) MRI of a model solution and a dried human molar at 9.4 T. A full 3D image dataset of the tooth was obtained, rich in contrast and similar to the resolution of standard cone-beam computed tomography.

## Introduction

The advent of fast sequences for magnetic resonance imaging (MRI) with echo times (TE) of the order of microseconds has opened up new, previously inaccessible applications that include the imaging of solids and nuclei whose signal decays too fast to be detected otherwise.

**Fig 1:**
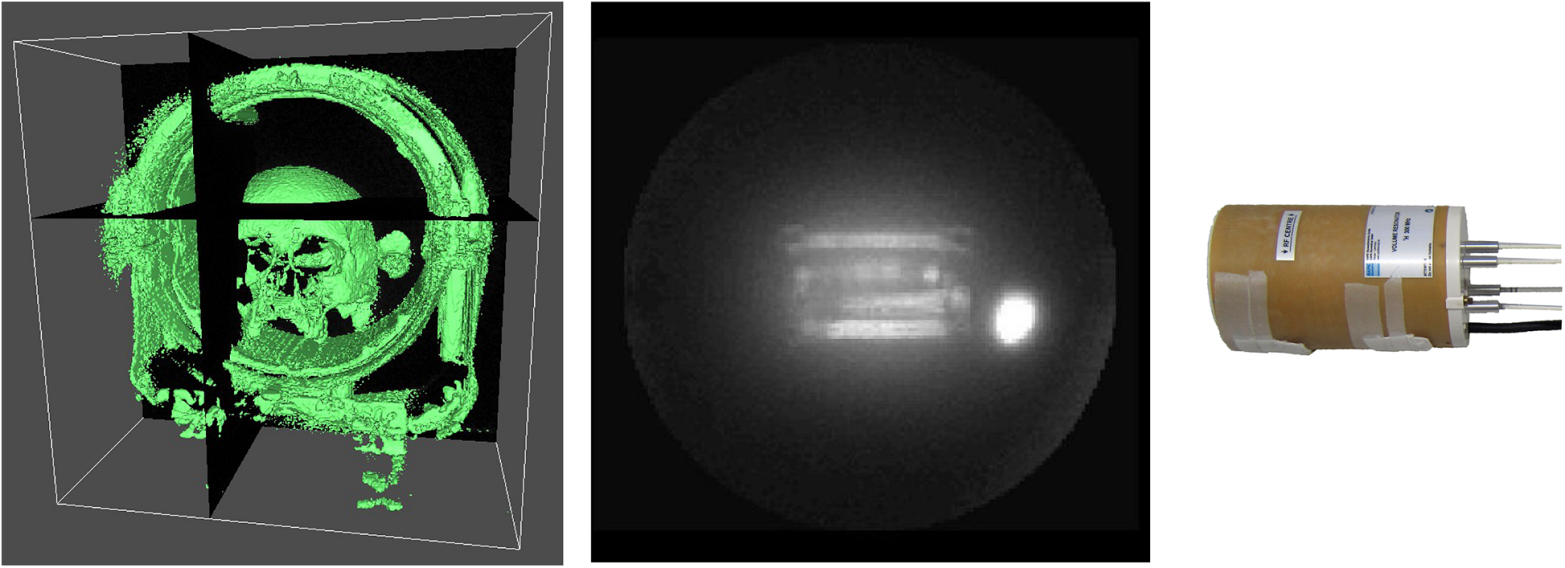
MRI of solids. 3D rendering of a skull found in a late roman settlement, estimated 300–400 A.D., and head coil acquired with an UTE sequence at 1.5 T (left). 3D maximum intensity projection of a ^1^H-ZTE image of a quadrature mouse coil at 9.4 T (center, QR_2_) and corresponding photograph (right).

In conventional MRI-sequences, the signal of solid-state or otherwise short-lived MR-active nuclei decays during the delay between signal excitation and its detection when the spatial information is encoded. In specialized MR sequences like ultra-short TE (UTE), zero-TE (ZTE) [1,2], sweep imaging with Fourier transformation (SWIFT) [3] and constant-time (CTI) or single-point imaging (SPI) [4,5], this delay can be shortened to a few micro seconds. As a consequence, many of the materials used for the construction of MR coils, patient or animal beds are readily depicted (**Fig 1**) [6]. This effect is not predominant if wet samples that are rich in signal are investigated, which is true for all *in vivo* applications. It is a major hindrance, however, if very dry samples are the subject of the examination, e.g. mummies [7–9], teeth [10–15], bone [16] or ceramics. Here, signal sources outside the imaging volume, the field of view (FOV), cause severe artifacts. One way to avoid these artifacts is to include all signal sources by increasing the FOV. Because this volume is typically much larger than the object of interest, the image resolution is reduced or the scan time increased.

Another solution is to avoid these signals in the first place by using e.g. hydrogen-poor materials for the construction of coil, bed and support. This approach was discussed before [6,12] and is the subject of this contribution.

Here, we present cost-efficient and very versatile single- and dual-tune transmit-receive coils of modular design with low hydrogen content. These coils offer a high and wide frequency range that allows multinuclear (e.g. ^1^H, ^19^F, ^17^O and ^23^Na) conventional and UTE/ZTE MRI. In contrast to other implementations, entirely plastic-free, self-supporting copper inductors were developed and used in conjunction with circuit boards with dramatically reduced hydrogen content.

The modular approach enabled a great flexibility with respect to the active volume, resonance frequency and thus nuclei. Conventional MR coils are one fixed and complete assembly that doesn't allow for changing the active volume or frequency. Instead, an entirely new coil is required, whose cost easily exceeds a few €/$ 10.000.

Our approach follows a different path that allows one to choose an appropriate circuit board and an appropriate self-supporting, copper-only inductor to assemble a coil that has the desired frequency and active volume without soldering.

We demonstrate the capability of these coils by acquiring high-resolution ^1^H and ^23^Na-ZTE images of model solutions, a dried mucsa domestica and a human tooth at 9.4 T. The low hydrogen content of the entire assembly allowed us to use a FOV that was smaller than the active volume of the coil, which was not feasible with the standard, hydrogen-rich commercial coils.

## Materials and Methods

### Construction of coils

To achieve a high flexibility and to reduce the associated cost and construction effort, we chose a modular approach to construct the coils, where the inductors and the circuit board (CB) were exchangeably mounted on holders made from polytetrafluroethylene (PTFE).

The complete coil assembly consisted of the inductor, removably connected to a circuit board, and a contraption to hold the assembly. All components were designed to have low hydrogen content.

#### Inductors

The inductivity and self-capacity of solenoidal coils with the desired inner volume of a few cm^3^ were found to be 10^2^–10^3^ nH and of the order of 1 pF. These values yield a self-resonance of the desired resonance frequency of the order of 10^2^ MHz (using *f* = (2 π √(LC)) ^-1^). With a tuning capacitor added, the desired resonance frequencies of 300 and 400 MHz could not be realized.

**Fig 2:**
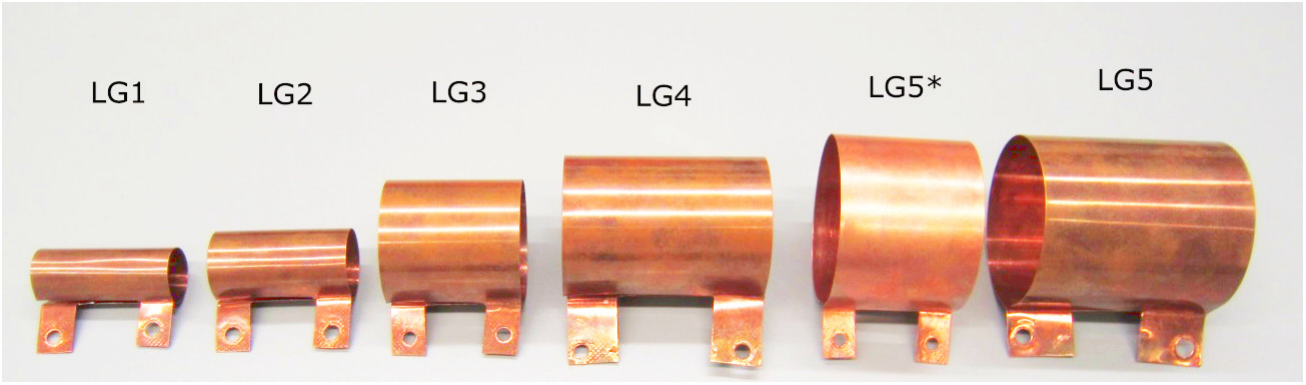
Representative photograph of several loop-gap inductors that were constructed (LG_1–6_). Note the ledges used for connection. Dimensions are provided in Table 2.

**Fig 3:**
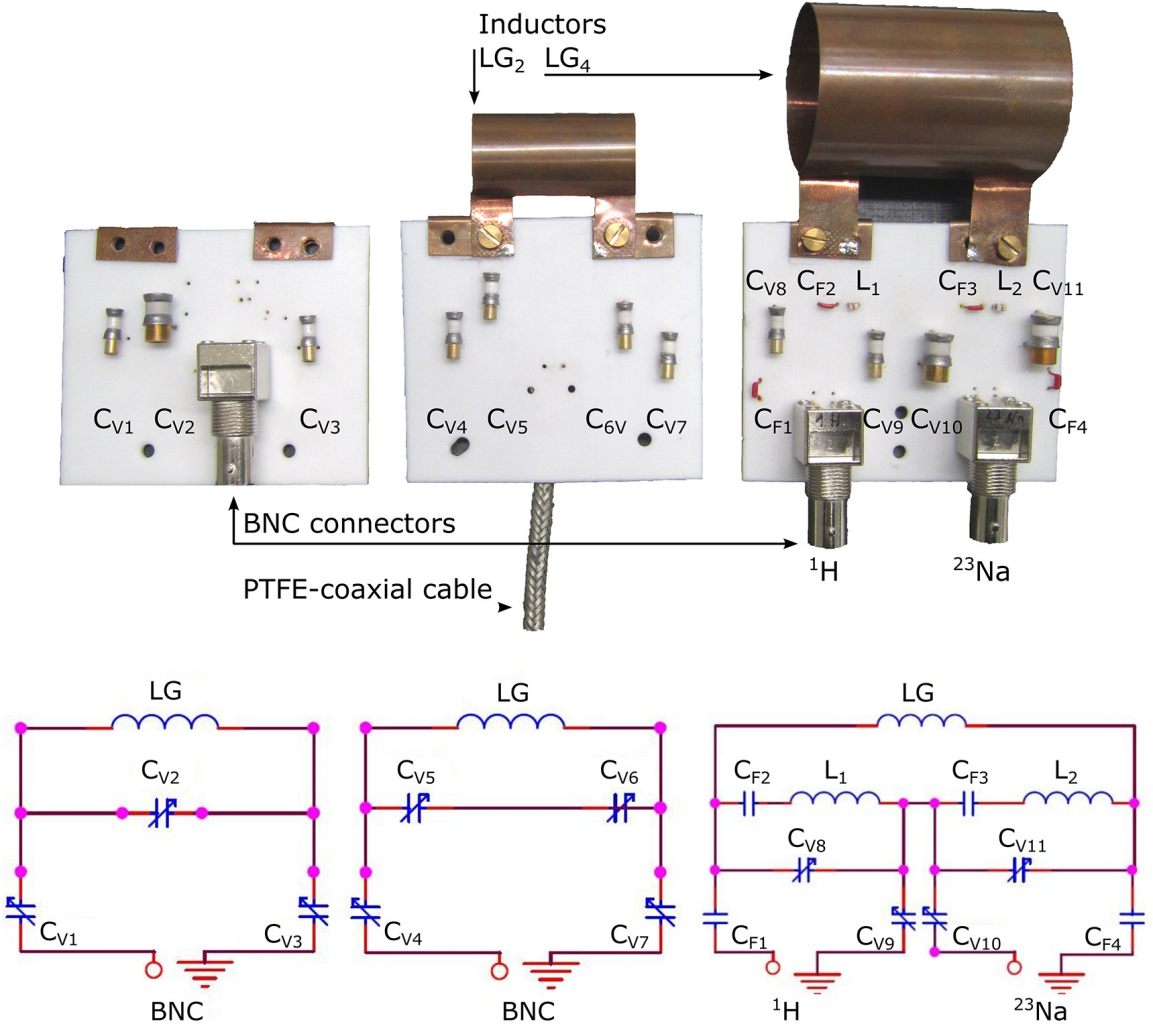
Photographs and schematics of the single-tune circuit boards (CB_1_, left and CB_2_, center) and dual-tune circuit board (CB_3_, right) with and without loop-gap (LG) inductors. Conventional BNC connectors (left and right) as well as a custom-made PTFE coaxial cable with low hydrogen content are shown (center). Values of variable capacitors are: C_V1,3-9_ = 0.3–3.5 pF, C_V2,10,11_ = 1.1–16 pF; fixed-value capacitors: C_F1_ = 1 pF, C_F2_ = 100 pF, C_F3_ = 6.8 pF, C_F1_ = 10 pF; inductors: L_1,2_ = 22 nH.

Loop-gap (LG) resonators of the same inner volume have a much lower inductivity of the order of 10^1^ nH. LG inductors were used for constant-time imaging (CTI) or single-point imaging (SPI) before, but at a lower, single frequency of 200 MHz, incorporated into a proton-poor plastic housing and with a small active volume [6]. Here, we used self-supporting loop-gap resonators that were manufactured in various sizes of copper (**Fig 2**, Table 1). Still, to reach the desired frequencies, it was necessary to use capacitors in a row to reduce the total capacitance, as shown in Fig 3 (all variable capacitors from Alfred Tronser GmbH, Germany, all fixed value capacitors from SRT Resistor Technology, Cadolzburg, Germany, all inductors from EPCOS AG, Munich, Germany).

**Table 1:** Quality factor (Q), tunable frequency range (*f*_r_) and addressable nuclei at 7 T and 9.4 T for coils composed of a circuit board (CB) and loop-gap (LG) resonator, in comparison to a commercial quadrature resonator (QR_2_) and linear resonator (LR). The proprietary connectors of QR_1_ did not allow a connection to the network analyzer.

CB	LG	Loading	*Q* ± SD (*f*(MHz))	*f*_*r*_ (MHz)	Nuclei
CB_1_	LG_2_	No	234 ± 13 (300)	114–462.5	^7^Li, ^31^P, ^3^He, ^19^F, ^1^H
		Yes	124 ± 4 (300)	113.4–461.8	
	LG_4_	No	89 ± 2 (300)	104.1–392.2	
		Yes	82 ± 2 (300)	104.1–391.9	
	LG_6_	No	176 ± 7 (300)	103.6–383.8	
		Yes	169 ± 7 (300)	103.6–383.6	
CB_2_	LG_2_	No	219 ± 9 (400)	365.6–666.3	^1^H, ^19^F
		Yes	193 ± 7 (400)	364.9–660.6	
CB_3_ Port 1	LG_4_	No	47.7 ± 0.8 (105.8)	88.3–131.7	^7^Li,^31^P, ^23^Na, ^13^C, ^1^H, ^19^F
		Yes	48.3 ± 0.8 (105.8)	88.3–131.7	
CB_3_ Port 2	LG_4_	No	46.5 ± 0.4 (400)	332.8–564.2	
		Yes	46.5 ± 0.4 (400)	332–564.2	
QR_2_		No	445 ± 35 (400)	392.6–416.8	^1^H
QR_2_		Yes	400 ± 30 (400)	392.5–416.4	
LR		No	42.5 ± 0.3 (400)	378.4–403.7	
LR		Yes	42.5 ± 0.3 (400)	378.4–403.6	

To facilitate the combination of various inductors of different sizes with several circuit boards without soldering, ledges were added to the loop-gap cylinders. These ledges were connected to matching copper terminals on the circuit boards using copper screws.

The construction of the inductors was facilitated by cutting thin copper sheets of 0.5 mm thickness to the required shape before preparing the ledges and bending the cylinders. Several inductors were constructed, some are shown (LG_1_ –LG_6_) and three were characterized in detail (LG_2_, LG_4_, LG_6_).

#### Circuit board

The circuit boards (CB) were cut from a slab of polytetrafluorethylene (PTFE) to slices of ≈ 2 mm thickness (Table 1. Variable and fixed capacitors were mounted on holes drilled through the PTFE board and connected with silver-plated wire following the scheme in Fig 3. A BNC connector (TE Connectivity, Greenpar, Germany) was mounted at a distance of ≈ 3 cm from the 5∙5∙2 mm copper terminals that held the inductors. The dual-tune circuit board (CB_3_) was equipped with two input/output ports.

A PTFE coaxial cable was soldered to the circuit board to replace the BNC jack and coaxial cable. The PTFE cable was constructed by removing the outer insulation layer of a non-magnetic coaxial RG58 cable, and replacing the insulation layer between outer and inner conductor by PTFE tubing with 4 mm outer and 2 mm inner diameter (FA. Bürkert, Germany).

For each assembly, the addressable frequency range was measured using a network analyzer in S11 mode (E5061B, Agilent Technologies, USA). Likewise, the Q-factor was determined with and without 5 ml of an aqueous model solution that contained 0.9% NaCl (phantom P1) by dividing the resonance frequency by the half width at half minimum of the S11-attenuation curve (Table 2).

**Table 2:** Dimensions of exchangeable loop-gap (LG) inductors that were mounted on the circuit boards (CB) and sensitive volume of the commercial quadrature birdcage resonators (QR_1_ and QR_2_) and linearly-polarized birdcage resonator (LR).

Object	Length (mm)	Diameter or width (mm)	Ledge length (mm)	Ledge width (mm)
LG_1_	40	15	15	10
LG_2_	40	20	15	11
LG_3_	40	36	15	10
LG_4_	60	42	20	15
LG_5_	40	52	15	15
LG_6_	60	51	16	20
CB_1_	6.6	7.9	-	-
CB_2_	7.0	7.5	-	-
CB_3_	6.5	8.2	-	-
QR_1_ (7 T)	60	42	-	-
QR_2_ (9.4 T)	59	37	-	-
LR (7 T)	112	74	-	-

#### Holder

A holder was manufactured from PTFE to mount the circuit board and inductors of various sizes. It was adapted to match the available animal beds of the MRI systems. The setup enabled precise placement of the samples within the center of the coil outside the bore instead of tedious placement of the sample within the bore-mounted standard coils.

### MRI, CBCT and phantoms

A 7 T and 9.4 T small-bore MR imaging system was used (Biospec 7/20 and 9.4/20, respectively, ParaVision 5.1, Bruker, Germany) in conjunction with the presented coils and a quadrature birdcage mouse coil for 7 T (QR_1_, Rapid Biomed, Würzburg, Germany), a quadrature birdcage mouse coil for 9.4 T (QR_2_, Bruker) as well as a linearly-polarized birdcage rat coil for 9.4 T (LR, Bruker). Product FLASH- as well as ZTE-sequences were applied with parameters indicated where appropriate.

**Fig 4:**
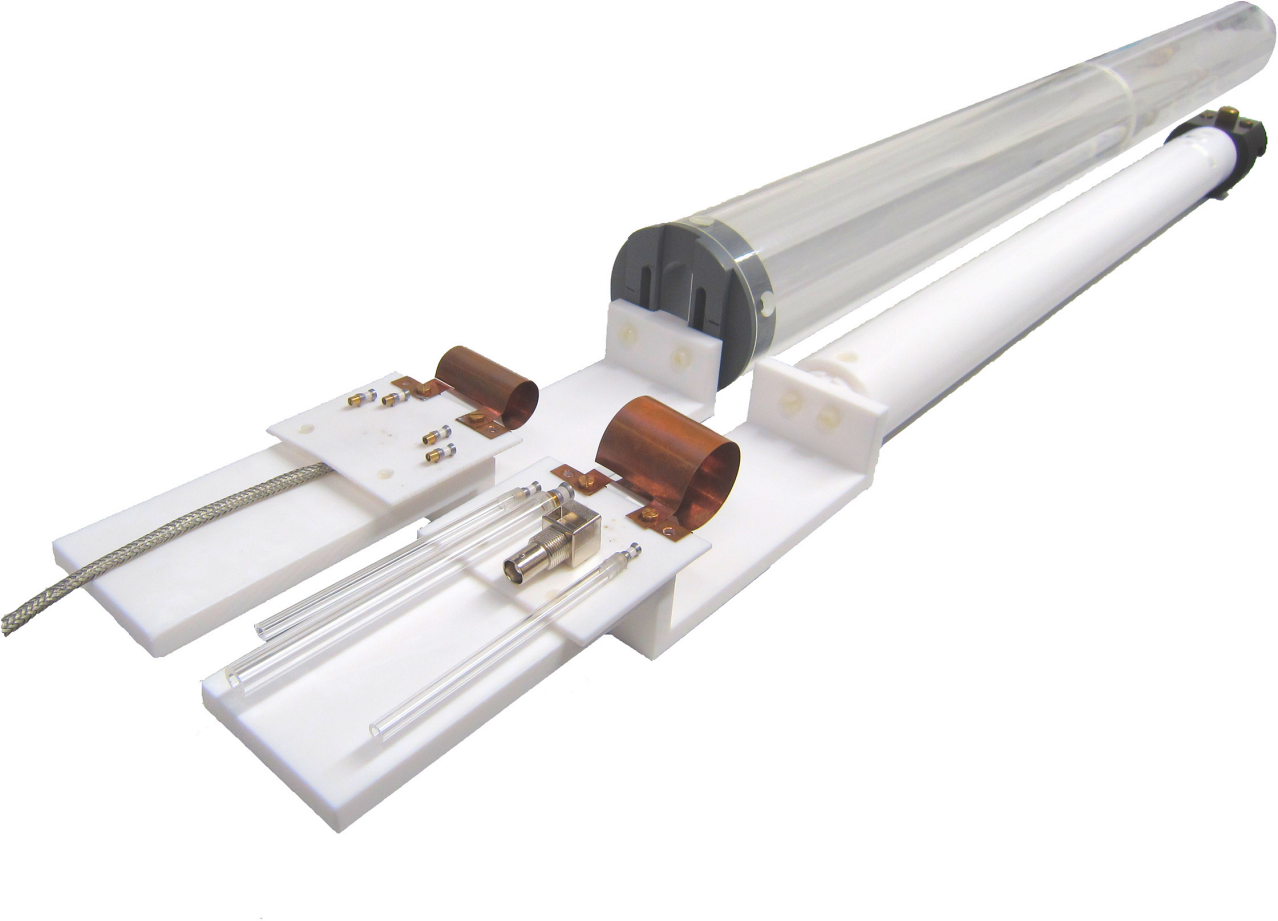
Photograph of the complete coil assemblies, CB_1_ with LG_4_ (front) and CB_2_ with LG_2_ (back), ready to be mounted to the bores of the 7 T and 9.4 T MR systems.

The coils were mounted on the MR system (Fig 4), loaded with a phantom and introduced to the bore, where they were tuned to the resonance frequency and matched to the impedance of the MR system. The manufacturer’s standard procedure to adjust transmitter frequency, flip angle, receiver gain and *B*_0_ homogeneity were applied, using a slice orthogonal to the phantom axis (Table 3). The calibration of the flip angle is expressed as the power that is required for a 90° block pulse of 1 ms length. Note that the cylindrical active volume of the commercial coils was in parallel to the magnetic field *B*_0_ and the LG resonators were perpendicular.

**Table 3:** Signal, noise, signal-to-noise-ratio (SNR) and reference pulse power (*P*_W_^90^) of a 1 ms, 90°-pulse for the coils composed of a circuit board (CB) and loop-gap (LG) inductor, as well as for commercial coils that were polarized in quadrature (QR_1_, QR_2_) and linear (LR).

Coil	*f* (MHz)	Signal (10^6^)	Noise (10^4^)	SNR	*P*_W_^90^ (W)
CB_1_ + LG_2_	300	1.11	1.03	107.8	1.13
CB_2_ + LG_2_	400	2.06	1.27	162.2	0.411
CB_2_ + LG_1_	400	5.14	1.5	342.7	0.103
QR_1_	300	1.85	0.68	271.7	0.143
QR_2_	400	5.9	1.05	561.9	0.530
LR	400	0.94	0.95	98.4	4.72

The manufacture’s specifications for maximum peak (5 ms) and mean power for QR_1_ were 200 W and 1.9 W, 400 W and 4 W for QR_2_, and 750 W and 22 W for LR, respectively.

**Fig 5:**
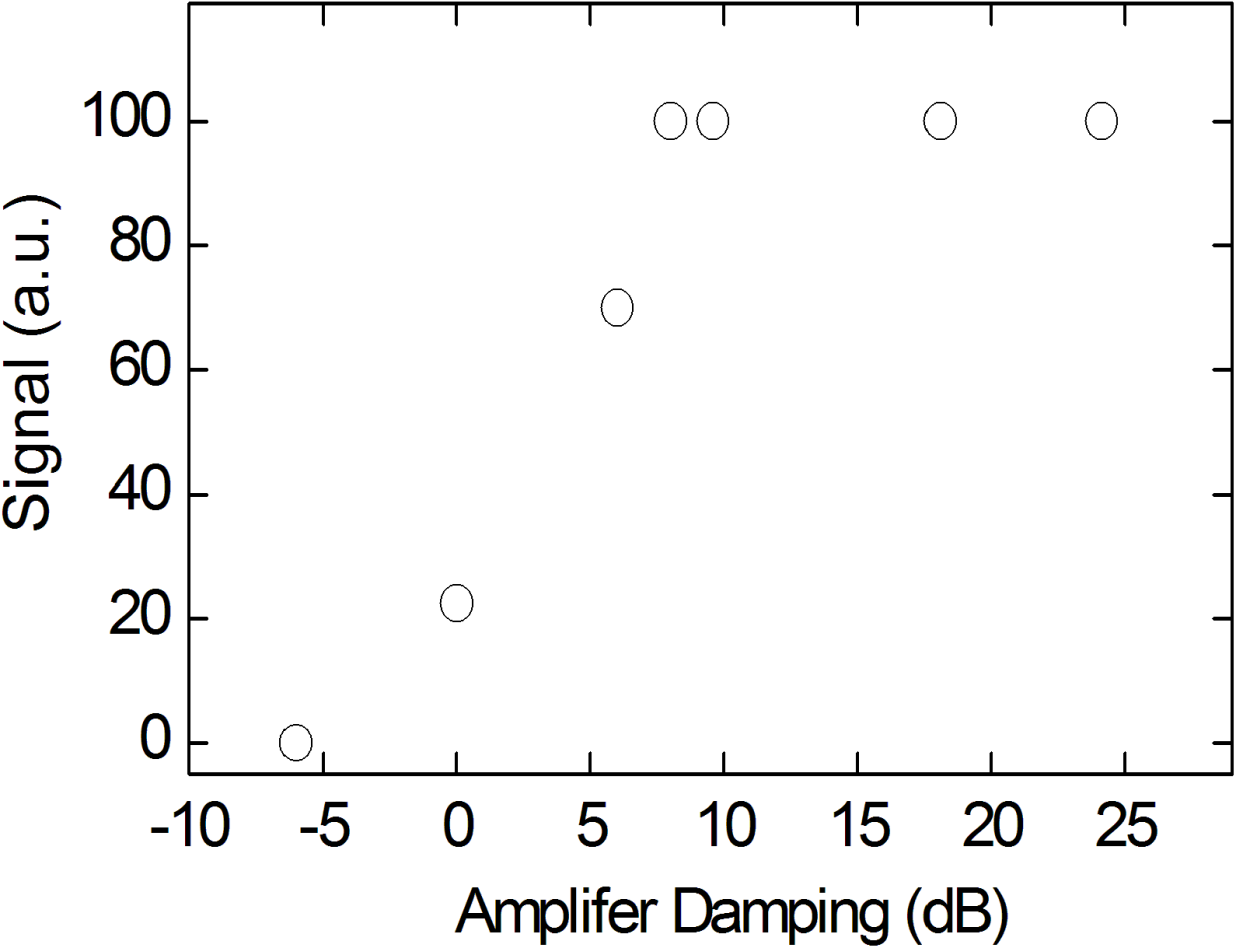
^1^H-MR signal height of a tooth acquired with unlocalized spectroscopy as a function of pulse power using a constant flip angle (α ≈ 6°) at 9.4 T with CB_2_ and LG_1_. Note that the MR signal decreased for powers exceeding 22 W.

To determine the maximum pulse power applicable to the constructed coils, non-localized ^1^H or ^23^Na spectra were acquired with a constant flip angle and variable pulse power *P*_W_ and pulse length. For example, the signal was found to decrease for a single-tune ^1^H coil that was composed of CB_2_ and LG_1_ at 9.4 T for a pulse power exceeding *P*w = 22 W (Fig 5), and for the ^23^Na-channel of the dual tune coil at 7 T at a pulse power exceeding 170 W.

The transmitter power *P*_W_ in Watt was obtained by converting the attenuation setting *A*_dB_ in dB using the manufacturer's calibration of the amplifier's maximum output at 50 Ωnamely 542 W for the 300 MHz ^1^H-channel at 9.4 T, and 835 W for the X-nuclei channel at ^23^Na-frequency of 105.8 MHz at 7 T (*A*_dB_ = -6 dB–10 ∙ log_10_(*P*_W_/542 W) dB).

The images were reconstructed using the manufacturer’s software and prepared for publication using open-source software (ImageJ [17], MIPAV [18], inkscape and GIMP (www.gimp.org). The signal-to-noise ratio (SNR) was obtained by dividing the mean signal of a region of interest (ROI) by the standard deviation of the noise from an apparently signal-free region of the image. The values were obtained using the manufacturer’s software (Paravision 5.1, Bruker).

The background signal of the constructed coils was investigated by applying a ZTE sequence to an empty coil at 9.4 T (TR = 4 ms, a = 3.3°, 100 kHz bandwidth, 8 cm FOV, 128^3^ matrix).

To compare the SNR among the coils, a FLASH sequence was used in conjunction with phantom P1 (TE = 6 ms, TR = 100 ms, α = 30°, 128 matrix, 6 cm FOV, 2 mm slice thickness; phantom P1: 5 ml solution of 0.9% NaCl in an Eppendorf tube).

To demonstrate MRI of dry samples and the associated artefacts, a dried musca domestica was examined with ZTE MRI at 7 T using CB_1_ and LG_2_ in comparison to QR_1_ (TR = 1 ms, α = 2°, FOV = (3 cm)^3^, matrix 64).

At 9.4 T, an extracted human molar was dried for a few hours before it was imaged with ZTE MRI using CB_2_ and LG_1_ (TR = 4 ms, α = 5.6°, BW = 200 kHz, FOV = 2.5 cm, Matrix 128, voxel size = 195 μm, 6 μs pulse at 10.8 W, N = 80, *t*_acq_ = 4.37 h). For comparison, a CBCT of the tooth was acquired afterwards (FOV 14.5 cm, nominal resolution 250 μm, 85 kV, 300 images, Scanora 3D, Soredex, USA). Informed consent was obtained from the donor for use in this study.

Multinuclear ZTE MRI of ^1^H- and ^23^Na nuclei was demonstrated without moving the setup using CB_3_ and LG_4_ in conjunction with a model solution of deionized water with 100 g NaCl / l (phantom P2, ≈ 12 ml, sequence parameters for ^1^H / ^23^Na: TR = 1 / 4 ms, α = 0.9°/4.5°, bandwidth 200 / 100 kHz, voxel size 0.78 / 1.56 mm, averages 1 / 2).

## Results

### ZTE MRI and background signal

**Fig 6:**
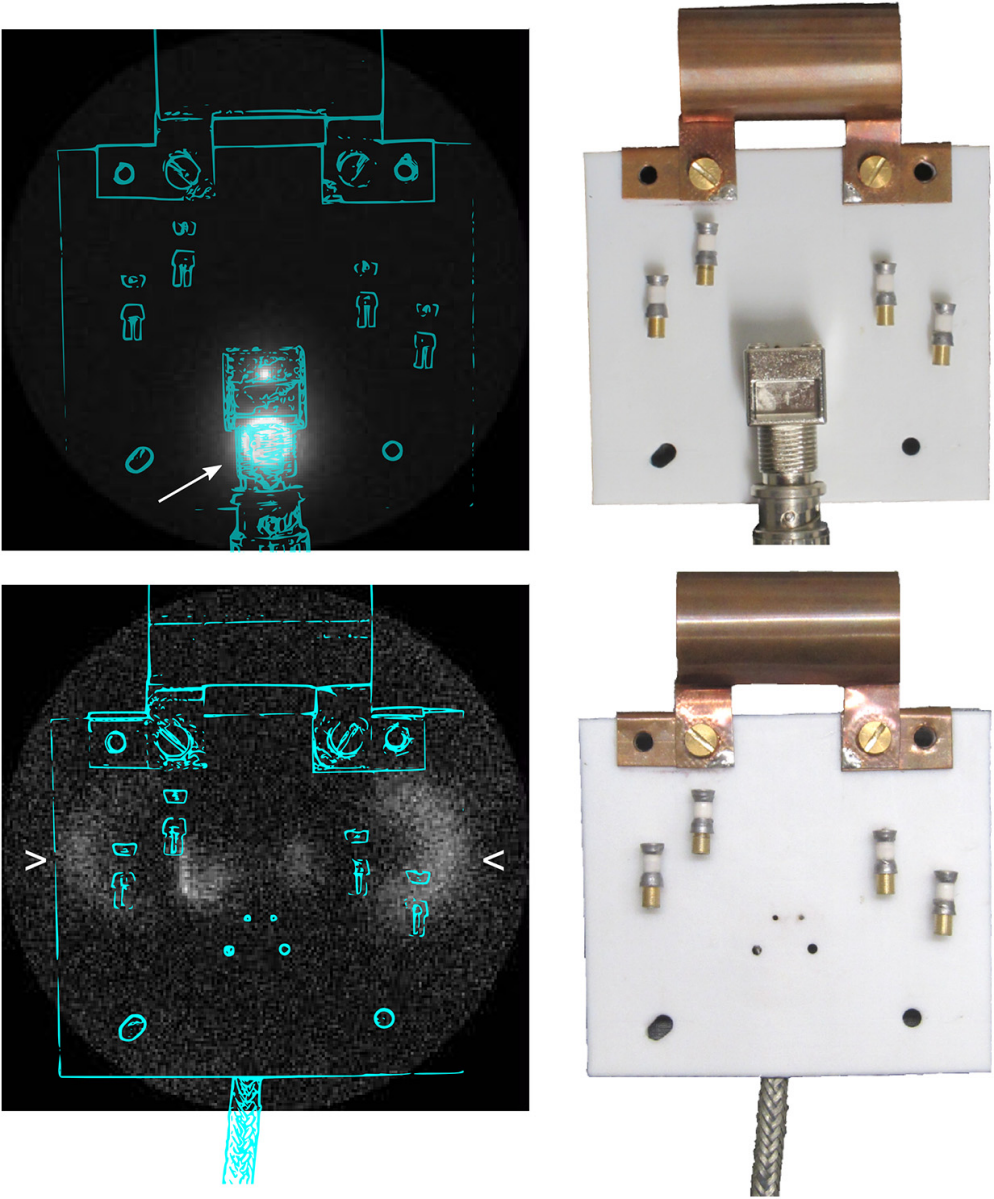
3D maximum-intensity-projection ZTE MRI and approximate coil positions (left) acquired at 9.4 T and photograph of coil assembled from CB_2_ and LG_2_ (right), either with a conventional BNC connector and coaxial cable (top), or a custom-made PTFE cable that was soldered to the circuit board (bottom). Note that the signal originating from the BNC connector was removed using the custom build cable. In turn, signal originating from the variable capacitors appeared close to the noise level (FOV = 20 cm).

All presented coils successfully enabled MRI at 7 T or 9.4 T, respectively. Interestingly, unwanted signal from the BNC jack and the connected coaxial cable was readily depicted on large-FOV ZTE images of the empty coil, despite the fact that both components were at ≈ 4 cm distance and thus well outside the inductor itself, as indicated in Fig 6, top, by the arrow. The signal vanished when the custom-made, hydrogen-poor coaxial cable was used instead as shown on the bottom of Fig 6. Instead, a new signal that originated from the approximate positions of the variable capacitors was observed, indicated by wedges.

**Fig 7:**
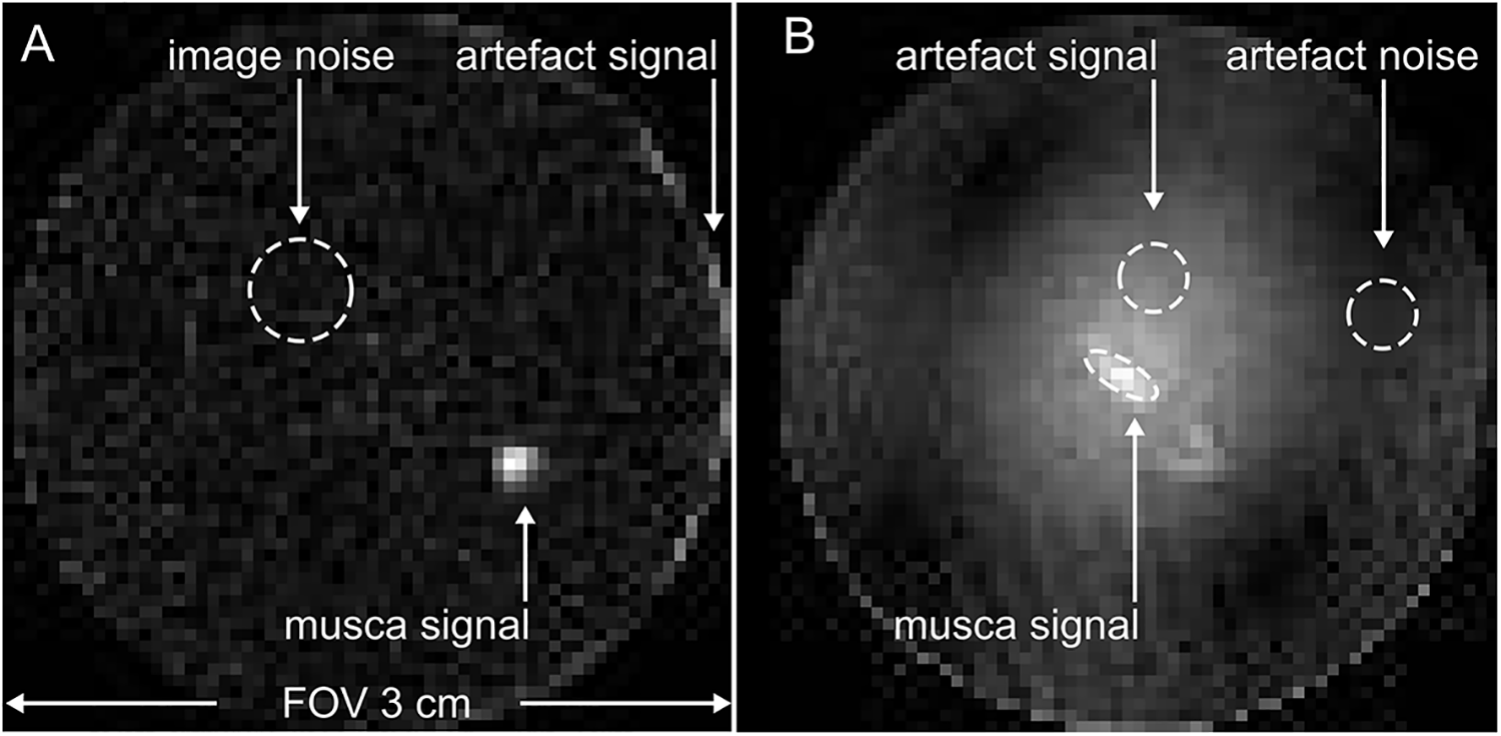
^1^H-ZTE images of a dried musca domestica acquired *ex vivo* with a commercial quadrature coil (left, QR_1_) and new, hydrogen-poor coil composed of CB_1_ and LG_2_ (right) with identical settings at 7 T. Note that the strong artifacts on the left were induced by signal sources from outside of the FOV that was (3 cm)^3^. In contrast, these artifacts did not appear when the modular loop-gap coil was used (right).

As it turned out, this signal was not sufficiently large to yield strong artifacts when the FOV was reduced to a volume within the inductor (Fig 7A). A musca domestica was depicted in one low-resolution ZTE acquisition with a SNR of 14, and only a thin, one-to-two-voxel artifact rim was visible at the edge of the FOV with a SNR of 7.5 (standard deviation (SD) of noise: 317 a.u.). In contrast, strong artifacts were observed across the entire FOV when QR_1_ was used with the same parameters (Fig 7B). Here, the diffuse distribution of the artifact exacerbated the accurate determination of the noise to calculate the SNR. Note that the artifact signal contributed to the region of noise and musca. With respect to the noise in a region where the artifact was less pronounced (SD = 1320 a.u.), the SNR was 27 for the mucsa domestica and 19 for the maximum artifact.

Note that the dried mucsa domesitca served as phantom with very low signal intensity only.

Whereas Horch *et al*. [6] reported MRI of ambient air moisture using a CTI sequence, we did not observe an increased signal from within the inductor.

### SNR and calibrations

The power required to apply a 1 ms, 90°-reference pulse with the presented coils was similar to the power required by the quadrature coils QR_1,2_, and less for the large linear coil LR (Table 3, e.g. 0.43 W for CB_2_ + LG_2_ and 0.54 W for QR_2_ at 9.4 T).

The SNR of the constructed coils was found to be of the same order of magnitude but lower with respect to the quadrature coils QR_1,2_, and higher with respect to the linear coil LR. This finding appears to be in contradiction to the principle of reciprocity but may, at least partially, be attributed to a higher noise figure of the LG coils. In contrast to the quadrature coils, the constructed coils weren't equipped with an outer r.f. shield.

### ^1^H and X-nuclei ZTE MRI

**Fig 8:**
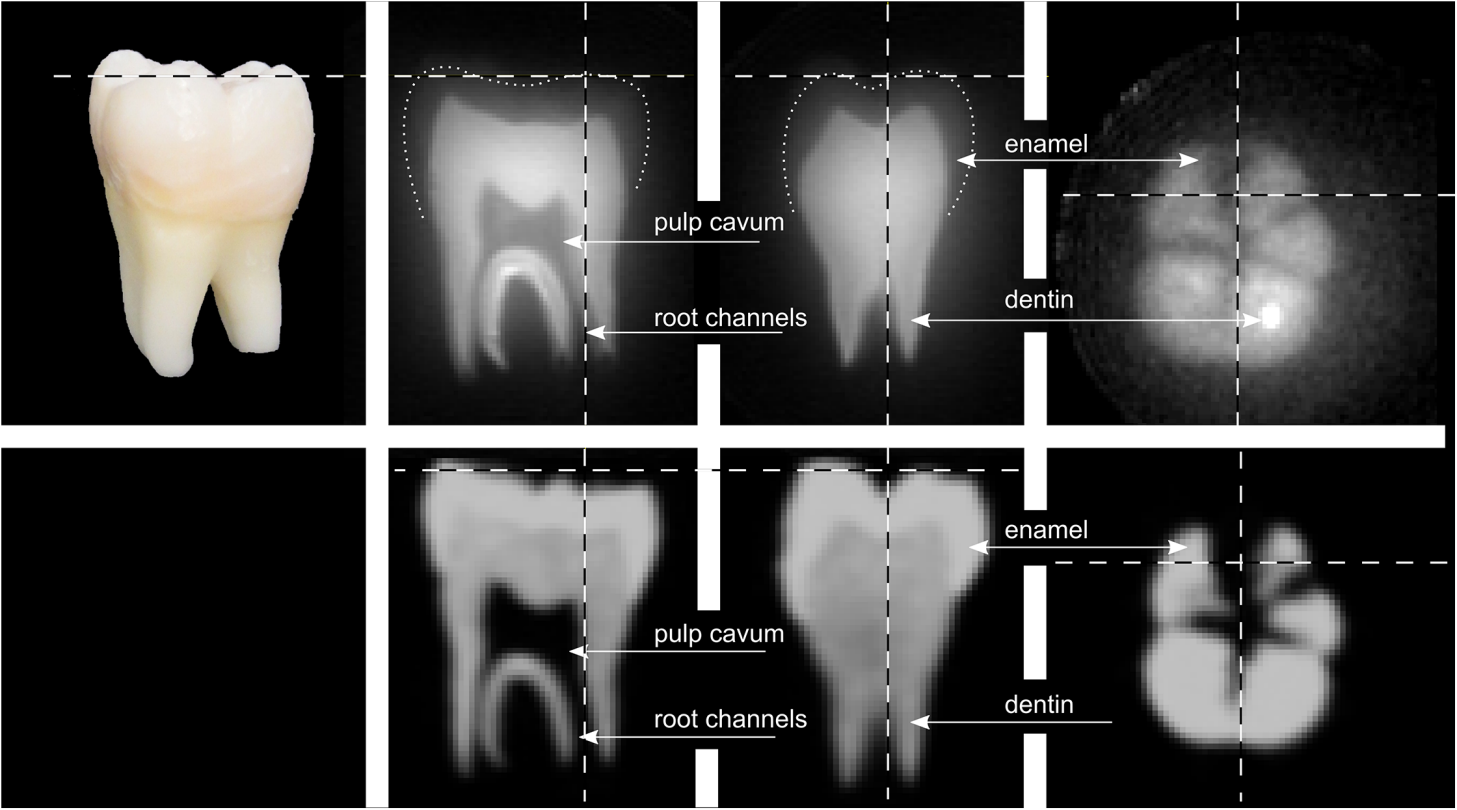
Photograph (top left), orthogonal reconstructions of a 3D ZTE MRI of an extracted human molar acquired at 9.4 T with CB_2_ and LG_1_ (top row) and 3D CBCT (bottom row). Note that the brightness of the MRI on the right was adjusted to show the enamel. Dashed lines indicate the slice positions, dotted lines the approximate outline of the enamel.

To exemplify the coils performance, an extracted human molar was subjected to ZTE MRI at 9.4 T using CB_2_ and LG_1_ (Fig 8). The FOV was set to (2.5 cm)^3^, the smallest volume covering the entire sample and well within the inductor. The shortest ^1^H-pulse feasible was 6 μs at 11.1 W, resulting in a ≈ 5.4° flip angle as calculated by the automatic calibration routine. At a matrix size of 128, an isotropic voxel size of (195 μm)^3^ was obtained and 80 averages were acquired (TR = 4 ms).

The pulpa cavum, dentin and, with the appropriate windowing, enamel were readily depicted. Before, a coil cooled to crygenic temperatures or much longer scan times were required to show enamel [12,13].

Compared to CBCT with a nominal resolution of 250 μm, the ZTE MRI appears sharper and richer in contrast, with the exception of enamel, which is much better depicted by CBCT.

**Fig 9:**
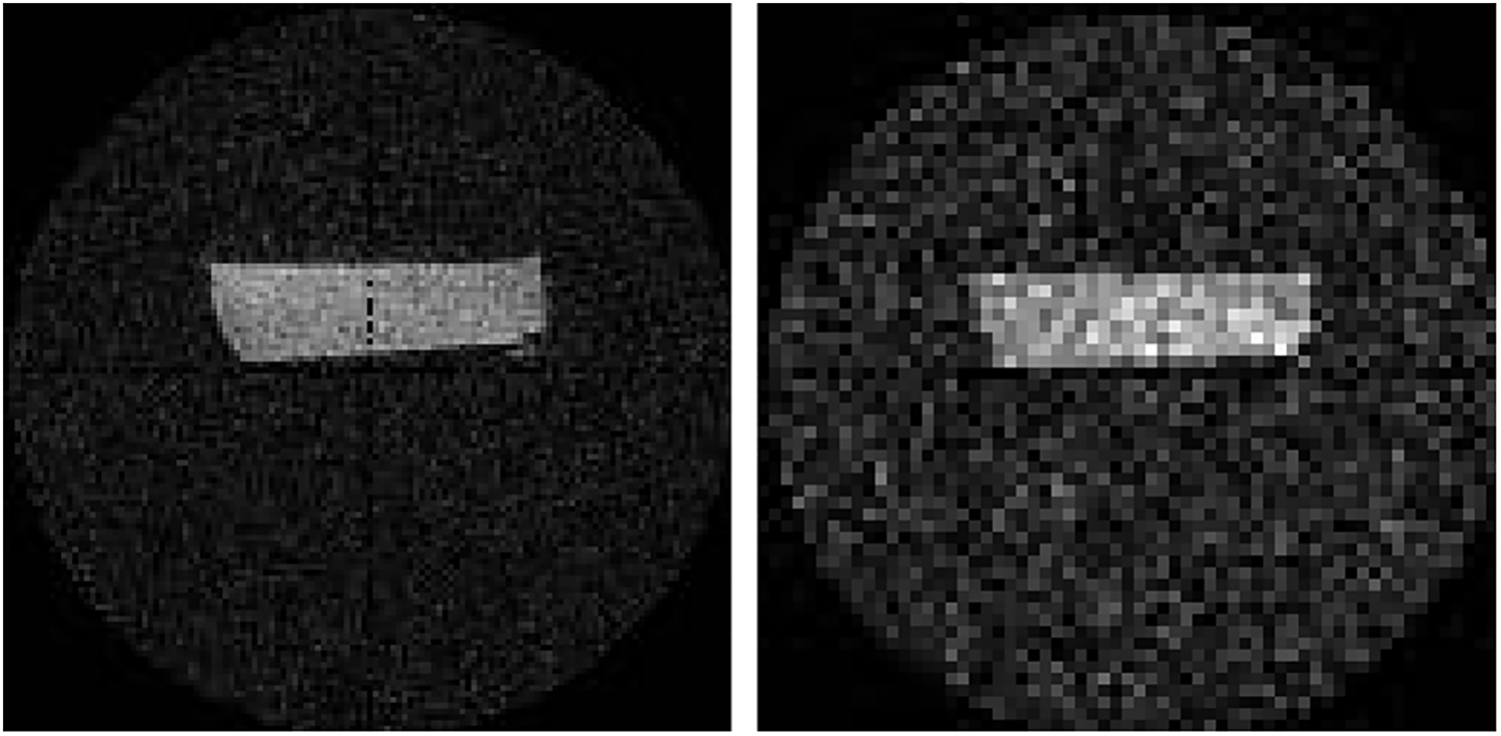
^1^H- and ^23^Na-ZTE MRI of an aqueous model solution containing 100 g NaCl / l acquired with LG_4_ and CB_3_ at 9.4 T.

To demonstrate the feasibility of multinuclear MRI, three-dimensional ^23^Na- and ^1^H-ZTE MRI of an aqueous NaCl solution was performed consecutively using the dual-tune coil (CB_3_, LG_4_) without moving sample. Both nuclei were readily depicted (Fig 9).

## Discussion

### Feasibility of multinuclear ZTE MRI

The hydrogen-poor transmit-receive coils with exchangeable inductors that were presented here are distinguished by the absence of all plastic materials in the vicinity of the inductor, the modular approach that allows an optimal filling factor and multinuclear applicability.

The coils are low in cost and relatively easy to manufacture, but, at the same time, provide satisfactory performance. Not surprisingly, inductors with a loop-gap geometry were successfully used for MRI of solids before [6]. However, the conceptual design presented here as well as the frequency, components and the sequences used are different.

All plastic materials near to the inductor were avoided by using a self-supporting copper inductor that was exchangeably mounted at a distance of one centimeter on a PTFE board. Other proton-rich materials including the insulation of a coaxial cable and a BNC terminal were removed. In the final setup, only the variable capacitors yielded ZTE signal, despite the fact that they were mounted outside of the inductor at a distance of several centimeters. Whereas the residual signal was too weak to cause interfering artifacts, it may be avoided in the future by using capacitors made from proton-poor materials only.

High resolution sodium and hydrogen images were acquired at 9.4 T to demonstrate the feasibility of ZTE MRI with the presented coils. It was the first time, to our knowledge, that dental enamel was depicted with a ZTE sequence using a coil at room-temperature. When the same sample was imaged using a conventional quadrature coil, strong artifacts arose that originated from the proton-rich components of the coil outside of the FOV.

### Geometry

Loop-gap resonators provide a small inductivity, a good *B*_1_ homogeneity but, given the geometry, no easy access to the inner coil volume when mounted in the magnet's bore.

This challenge was overcome in the current implementation by moving the entire coil assembly, mounted on the animal bed, in- and out of the magnet. In fact, this scheme proved to be beneficial when it came to placing a small sample accurately in the center of the coil. If sample placement outside of the bore is not an option, a simple saddle-shaped inductor that is connected to the presented circuit boards may constitute a viable alternative. It is expected, though, that the inductivity of a saddle-shaped inductor is higher than a comparable loop-gap resonator and that it will thus affect the resonance frequency.

### SNR

Both reference pulse power as well as SNR was similar or of the same order of magnitude for the commercial quadrature coils and constructed loop-gap resonators, respectively.

The differences in SNR that were found are not unexpected because of different geometries, materials and linear / quadrature polarization that is expected to yield √2 more SNR.

It appears though, that the somewhat lower SNR of the LG coils may be attributed to a higher noise, which may be attributed to relatively low-priced variable capacitors and the absence of a r.f. shield as was included in the commercial coils.

### Cost, flexibility and ease of use

The complete materials for one single-tune coil assembly with several inductors do not exceed €/$ 50, which is several orders of magnitude less than the cost of a commercial coil. However, as pointed out above, a higher SNR may be achieved with capacitors of higher quality that are more expensive and appropriate shielding. The matching and tuning of the coils in the bore of the magnet could be facilitated by long rods permanently connected to the capacitors, and a smaller range of the variable capacitors.

The wide tuning range of the capacitors enabled to image several nuclei with one setup, which greatly facilitates proof-of-principle X-nuclei experiments without the need of acquiring a dedicated coil whose cost easily exceed €/$ 10.000. The modular approach of using interchangeable inductors and circuit boards has proven to add significantly to the applicability and flexibility of the presented coils.

## Conclusion

Single and dual-tune coils for conventional MRI as well as close-to-zero echo-time MRI of ^1^H and X-nuclei at 7 T and 9.4 T were presented. Background signal was significantly reduced by constructing the coils from materials with very low hydrogen content using self-supporting plastic-free inductors and a coaxial cable with low hydrogen content. A modular approach enabled the combination of inductors of various sizes with one circuit board to assure an optimal filling factor, a wide range of addressable nuclei and a reduction of the overall cost to ≈50$. These properties enabled high-resolution ZTE MRI of solids with a FOV within the inductor as exemplified by imaging an extracted human tooth including its enamel.
