# Correction: Modular Coils with Low Hydrogen Content Especially for MRI of Dry Solids

**DOI:** 10.1371/journal.pone.0151505

**Published:** 2016-03-09

**Authors:** Timon Eichhorn, Ute Ludwig, Elmar Fischer, Jens Gröbner, Michael Göpper, Anne-Katrin Eisenbeiss, Tabea Flügge, Jürgen Hennig, Dominik von Elverfeldt, Jan-Bernd Hövener

Fig 5 appears incorrectly in the published article. Please see the correct Fig 5 and its legend here.

**Fig 5:**
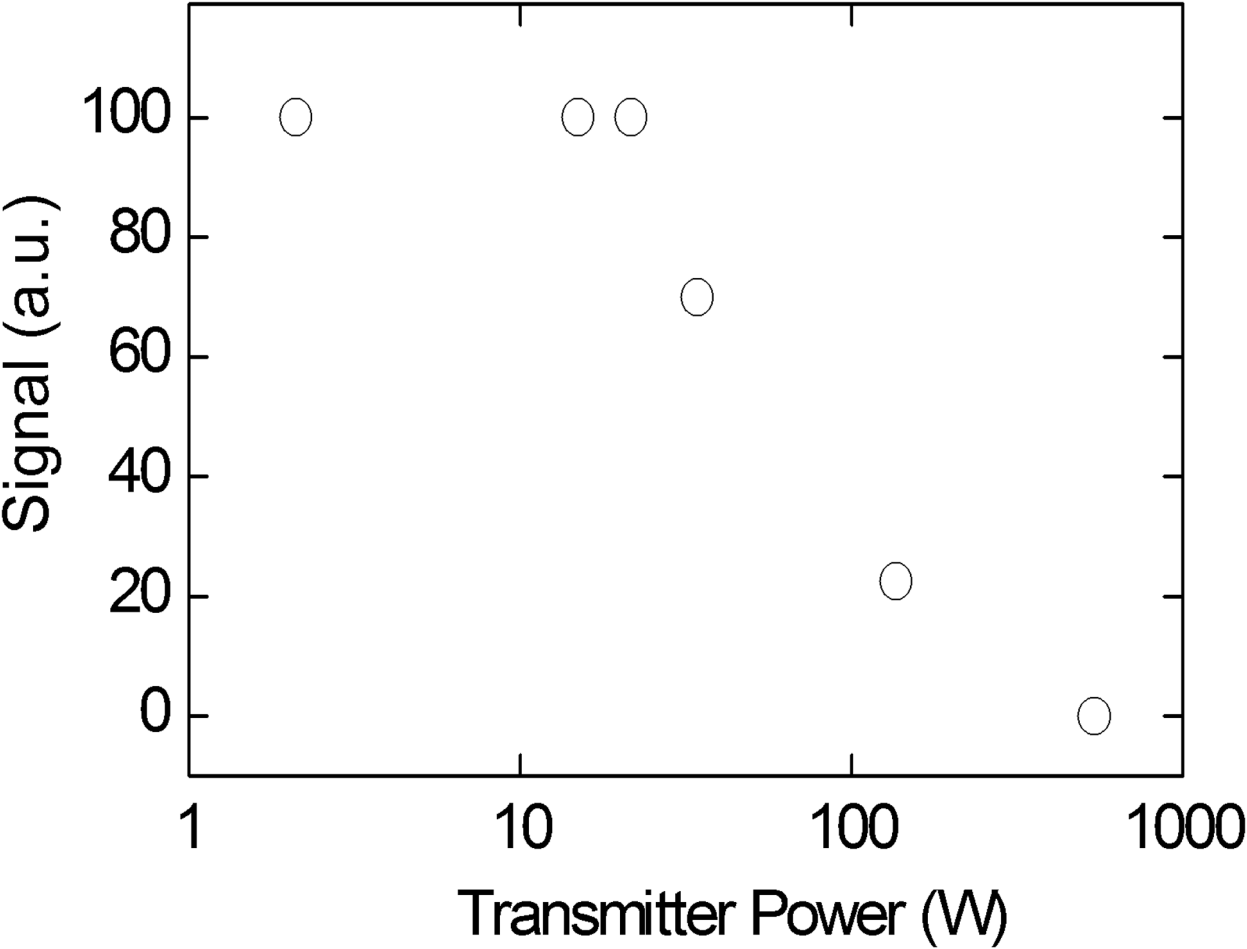
^1^H-MR signal height of a tooth acquired with unlocalized spectroscopy as a function of pulse power using a constant flip angle (α ≈ 6°) at 9.4 T with CB_2_ and LG_1_. Note that the MR signal decreased for powers exceeding 22 W.

## References

[1] Eichhorn T, Ludwig U, Fischer E, Gröbner J, Göpper M, Eisenbeiss A-K, et al (2015) Modular Coils with Low Hydrogen Content Especially for MRI of Dry Solids. PLoS ONE 10(10): e0139763 doi: 10.1371/journal.pone.0139763 26496192 10.1371/journal.pone.0139763 PMC4619699

